# Ion-pair pinning on perovskite quantum dots for high-efficiency air-processed light-emitting diodes with Rec. 2020 compliance

**DOI:** 10.1038/s41377-026-02247-z

**Published:** 2026-03-06

**Authors:** Yuhang Cui, Danlei Zhu, Jiawei Chen, Shuyue Dong, Yuanzhuang Cheng, Xiangyu Liu, Xinghua Yan, Zicong Jin, Lian Duan, Jian Xu, Dongxin Ma

**Affiliations:** 1 Key Lab of Organic Optoelectronics and Molecular Engineering of Ministry of Education, Department of Chemistry, Tsinghua University, 100084 Beijing, China; 2 MIIT Key Laboratory of Advanced Display Materials and Devices, School of Materials Science and Engineering, Nanjing University of Science and Technology, Nanjing, 210094 China; 3 State Key Laboratory of Flexible Electronics Technology, Tsinghua University, 100084 Beijing, China; 4 School of Interdisciplinary Science, Beijing Institute of Technology, 100081 Beijing, China

**Keywords:** Lasers, LEDs and light sources, Electronics, photonics and device physics

## Abstract

Perovskite quantum dot light-emitting diodes (QLEDs) offer superior efficiency and high colour purity, making them promising candidates for next-generation lighting and display technologies. However, fabricating the emissive perovskite quantum dot (QD) layer typically requires a protective atmosphere due to its air sensitivity, thereby increasing production costs and limiting industrial scalability. Here, we propose an ion-pair pinning strategy by using tetraalkylammonium triflate (NR_4_OTf) to enable ambient-air processing of formamidinium lead bromide (FAPbBr_3_) QD films. The trifluoromethanesulfonic acid anions (OTf^−^) hydrogen bond with FA^+^, inhibiting its detachment and passivating the uncoordinated Pb^2+^, while the tetraalkylammonium cations (NR_4_^+^) serve as X-type ligands to inhibit deprotonation. This dual ion-pair pinning effect stabilises the QD lattice and provides surface resistance to moisture and oxygen, thereby improving the uniformity, stability, and optoelectronic performance of air-processed QD films. The as-constructed air-processed QLED achieves a high external quantum efficiency (EQE) of 21.3% and a peak luminance of over 3 × 10^4 ^cd m^−2^ at 529 nm with Rec. 2020 compliance (EQE of 23.9% and luminance of over 8 × 10^4 ^cd m^−2^ for the N_2_-processed QLED). Our work eliminates the reliance on inert gas protection in perovskite QLED fabrication, laying a foundation for their low-cost, large-scale manufacturing and expansion into diversified applications.

## Abstract (web summary)

An ion-pair pinning strategy is proposed for air-processed FAPbBr_3_ quantum dot light-emitting diodes with Rec. 2020 compliance, eliminating inert gas dependence for low-cost scalable manufacturing.

## Graphical abstract

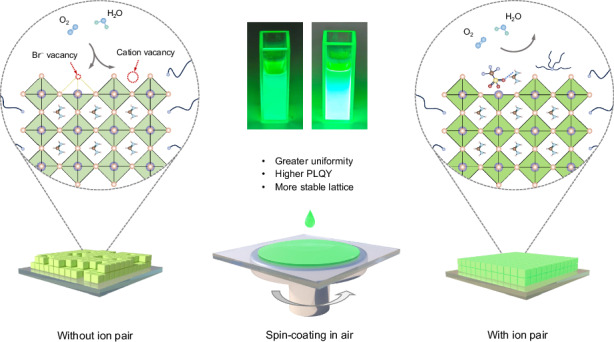

## Introduction

Perovskite quantum dots (QDs) show great promise for ultra-high-definition display applications due to their broad colour gamut coverage, high photoluminescence quantum yields (PLQYs), and narrow emission spectra^1–4^. In recent years, perovskite quantum dot light-emitting diodes (QLEDs) have made remarkable progress, achieving luminance over 10^5^ cd m^−2^ and superior external quantum efficiencies (EQEs) over 25%^5–9^. However, perovskite QLEDs face critical challenges in industrialisation because their active QD layers suffer environmental sensitivity and require inert gas protection^10,11^.

Formamidinium lead bromide (FAPbBr_3_) QDs exhibit pure green emission and are compatible with room-temperature synthesis, attracting considerable interest for broad colour gamut display^12–14^. Nevertheless, FAPbBr_3_ QDs are extremely sensitive to ambient air. During air processing, the moisture and oxygen would lead to loss of the organic cation and collapse of the inorganic framework, thereby worsening the properties of the perovskites^15,16^. Specifically, H_2_O molecules could hydrogen bond with FA^+^ and dissociate it from the perovskite lattice, and O_2_ molecules could induce deprotonation reactions of FA^+^ through photo-induced superoxide. Furthermore, collapse of the crystal structure could lead to halogen ion migration and conversion into halogen species, resulting in irreversible degradation of the perovskite structure and consequent performance loss. In addition, FAPbBr_3_ QDs also suffer from ligand-related issues. The commonly used ligands, octylamine (OTAm) and oleic acid (OA), are prone to deprotonate and detach from the surface of QDs due to their dynamic and weak interactions, resulting in abundant defects and undesirable ripening of QDs^17,18^. These issues are further exacerbated during air processing. During spin coating in ambient air, the QDs would expose a large specific surface area and undergo rapid solvent removal, making them highly susceptible to moisture- and oxygen-induced degradation^19^. Consequently, fabricating high-quality perovskite QD films under ambient conditions is challenging, hindering the industrial advancement of QLEDs.

In this work, we reported an ion-pairing pinning strategy by introducing tetraalkylammonium triflate into the precursor to pin on the surface of FAPbBr_3_ QDs, thereby achieving high-quality air-processed QD films. We demonstrated that the trifluoromethanesulfonic acid anions (OTf^−^) would hydrogen bond with FA^+^, inhibiting its detachment and passivating the uncoordinated Pb^2+^. Meanwhile, the tetraalkylammonium cations (NR_4_^+^), acting as X-type ligands, would inhibit deprotonation and form strong interactions with the surface of QDs. These pinning interactions could passivate QD surface states, improve QD stability, and suppress the nonradiative recombination, enabling the air-processing of high-quality QD films. The corresponding air-processed device showed a high EQE of 21.3% and a peak luminance of 30,683 cd m^−2^, representing the best performance of the reported air-processed perovskite light-emitting diodes (LEDs). The green electroluminescence (EL) peak was observed at 529 nm with a full width at half maximum (FWHM) of 21 nm, corresponding to the Commission Internationale de l’Eclairage (CIE) coordinates of (0.19, 0.76), which met the requirements of the Rec. 2020 standard. Our work represented a breakthrough in the industrialisation of low-cost and high-performance perovskite LEDs, expanding the potential of perovskite QDs for broader optoelectronic applications.

## Results

### Ion pair selection

We synthesised FAPbBr_3_ QDs in dimethyl formamide (DMF) with or without ion-pair pinning via a modified ligand-assisted reprecipitation (LARP) method under ambient conditions (25 ± 2 °C, 25% relative humidity), as detailed in the “Methods”. To identify the optimal ion pair, we selected a series of ion pairs, including NH_4_Br, TEABr (TEA^+^ denotes the tetraethylammonium cation), TBABr (TBA^+^ denotes tetrabutylammonium cation), NH_4_MeS (MeS^−^ denotes the methanesulfonate anion), and NH_4_OTf, and evaluated their effects on the PLQY of both QD solutions and air-processed QD films (Fig. S1). For the pristine QDs, air processing led to a large reduction in film PLQY (from 77 to 20%). In contrast, all ion pairs enhanced both QD solutions and air-processed QD films. Among the cations, TBA^+^ yielded the highest PLQY in air-processed QD films, whereas among the anions, OTf^−^ proved more effective. Therefore, we selected TBAOTf as the optimal additive (labelled as “target”).

**Fig. 1:**
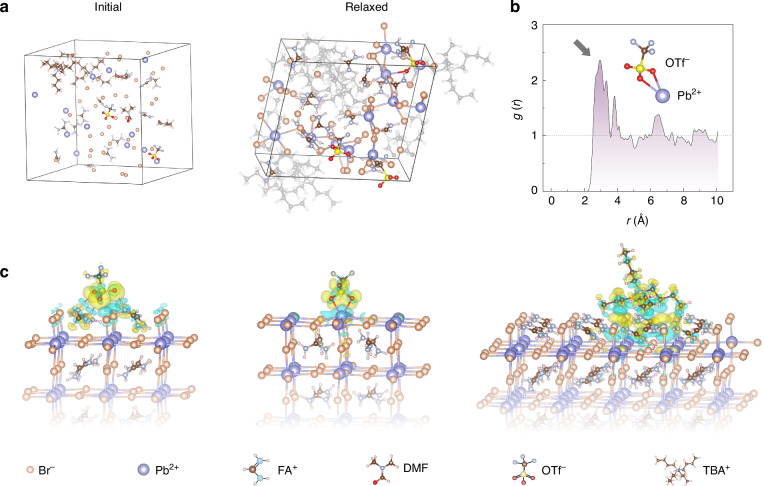
Theoretical calculations of the ion-pair pinning effect. **a** Initial and relaxed configurations of the precursor solution model, obtained from AIMD simulations. In the relaxed structures, only Pb^2+^, FA^+^, Br^−^, and OTf^−^ are highlighted for clarity; all other ions and solvent molecules are rendered transparent. **b** Radial distribution function *g*(*r*) between the O atoms of OTf^−^ and Pb^2+^, computed from the last 4 ps of the AIMD trajectory under the NVT ensemble. **c** Charge density difference plot showing the pinning of OTf^−^ onto the FABr-terminated and PbBr_2_-terminated perovskite surface, and TBA^+^ onto the FABr-terminated perovskite surface (blue, charge depletion; yellow, charge accumulation)

To validate our hypothesis regarding the role of ion-pair additives, we first conducted a series of density functional theory (DFT) calculations. Specifically, ab initio molecular dynamics (AIMD) simulations were employed to elucidate the crystallisation behaviours of FAPbBr_3_ QDs in the presence of TBAOTf. The simulation system consisted of Pb^2+^, FA^+^, TBA^+^, Br^−^, OTf^−^, and DMF molecules, which were initially distributed randomly (Fig. 1a). All simulations employed the exchange-correlation functional with Goedecker–Teter–Hutter (GTH) pseudopotentials and double-zeta valence plus polarisation (DZVP-MOLOPT) basis sets. The simulation protocol included a 9 ps equilibration under a constant-pressure and constant-temperature (NPT) ensemble, followed by a 10 ps run in the constant-volume and constant-temperature (NVT) ensemble. The temperature was maintained at 300 K, and the pressure was fixed at 1 atm. Upon reaching equilibrium, OTf^−^ was observed to consistently form hydrogen bonds with FA⁺ and strongly coordinate with Pb^2+^ in the precursor solution. The radial distribution function (*g*(*r*)) (Fig. 1b) exhibited a distinct first peak at *r* ≈ 2.5 Å, confirming that oxygen atoms from OTf^−^ lie within the first coordination shell of Pb^2+^, indicative of direct coordination interactions. These findings suggested that OTf^−^ could participate in modulating the precursor structure and crystallisation kinetics. This was potentially beneficial to the formation process of high-quality QDs, as OTf^−^ could restrict precursor diffusion through multiple interactions and promote the formation of monodisperse QDs^20^.

We then used static DFT calculations to investigate how the TBAOTf additives interact with FAPbBr_3_ QD surfaces (Fig. 1c). The optimised perovskite surface models were constructed based on both FABr-terminated and PbBr_2_-terminated (001) slabs, where FA^+^ sites were exposed on the FABr-terminated surfaces, whereas undercoordinated Pb^2+^ sites were exposed on the PbBr_2_-terminated ones. Charge density difference plots revealed that OTf^−^ would hydrogen bond with FA^+^ and establish strong coordination interactions with undercoordinated Pb^2+^ on the perovskite surface. In these plots, regions of charge accumulation were shown in yellow, while charge depletion was depicted in blue. These results indicated electron redistribution upon additive binding, confirming the active involvement of OTf^−^ in surface passivation. Furthermore, TBA^+^ exhibited binding affinity to the FABr-terminated surface, effectively inhibiting the detachment of FA^+^ from the perovskite lattice. This binding behaviour contributed to enhanced surface integrity and improved the resistance of QDs to environmental factors such as moisture and oxygen.

These theoretical results demonstrated that the two components of TBAOTf would act synergistically in regulating the crystallisation and enhancing the surface stability of FAPbBr_3_ QDs. The OTf^−^ anion would facilitate surface passivation through hydrogen bonding and coordination with Pb^2+^, while the TBA^+^ cation would anchor to the surface and prevent A-site cation (FA^+^) loss. This dual mechanism would collectively improve QD quality and environmental robustness.

### Ion-pair pinning effect on QD solutions

**Fig. 2:**
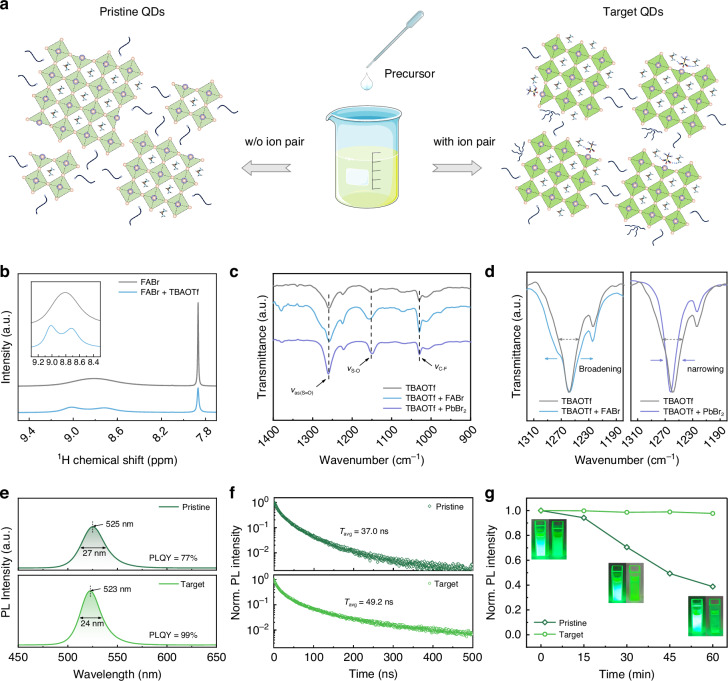
Ion-pair pinning effect on QD solutions. **a** Schematic illustration of the ion-pair pinning effect on QD solutions. **b** ^1^H NMR spectra of pure FABr and FABr + TBAOTf. The inset shows an enlarged view of a specific chemical shift region (9.2 – 8.4 ppm). **c** FTIR spectra of pure TBAOTf, TBAOTf + FABr, and TBAOTf + PbBr_2_. **d** Localised magnification of FTIR spectra. **e** PL spectra. **f** TRPL curves. **g** Stability of the QD solutions with the inset of photographs under 365 nm UV excitation light (right: pristine QDs; left: target QDs)

Based on the theoretical analysis, we expected that TBAOTf could regulate the crystallisation of QDs through multiple interactions, to yield QDs with enhanced PLQYs (Fig. 2a). Probing the interaction between TBAOTf and the perovskite precursor components—a prerequisite for the surface pinning effect—we employed nuclear magnetic resonance (NMR) and Fourier transform infrared (FTIR) measurements. In the ^1^H NMR spectrum of pure FABr, we observed a sharp peak at 7.87 ppm, corresponding to the α-hydrogen, as well as a broad peak at around 8.8 ppm, attributed to the resonance of N-H active hydrogen in FA^+^. With the addition of TBAOTf, the broad peak showed signs of splitting. This indicated that TBAOTf could change the localised chemical environments of two types of N-H in FA^+^, suggesting the hydrogen bonding between FA^+^ and TBAOTf (Fig. 2b)^21^. In the FTIR spectra of pure TBAOTf, the peaks at 1030 cm^−1^, 1153 cm^−1^, and 1258 cm^−1^ were attributed to the stretching vibration of the C-F bond (*ν*_C-F_), the symmetric stretching vibration of the S-O bond (*ν*_S-O_), and the asymmetric stretching vibration of the S = O bond (*ν*_as(S=O)_), respectively (Fig. 2c). From the enlarged view of Fig. 2d, the addition of FABr caused a slight decrease in wave number and peak broadening of *ν*_as(S=O)_, which showed the hydrogen bonding formation between OTf^−^ and FA^+^ (ref. ^12^). With the addition of PbBr_2_, the wavenumber of *ν*_S-O_ decreased, while that of *ν*_as(S=O)_ increased and the peak narrowed. These results could be attributed to the coordination of the sulphonate group in OTf^−^ with Pb^2+^ (refs. ^22–24^). These observed interactions were consistent with DFT calculations (Fig. 1a, b), providing experimental evidence that the ion pairs could participate in the QD crystallisation process.

We then measured the optical properties of the QD solutions. Compared to the pristine QD, the target QD solution exhibited blue-shifted emission with a smaller FWHM and a higher PLQY (Fig. 2e and Fig. S2). The superior optical performance of the target QDs could be attributed to their improved structural properties, resulting from the participation of ion pairs in QD crystallisation. Transmission electron microscopy (TEM) revealed a more concentrated size distribution for the target QDs, whereas the pristine counterparts showed a broader distribution, with many aberrantly large or small particles (Fig. S3). This polydispersity in the pristine QD size might lead to unnecessary self-absorption and energy transfer, broadening the PL and lowering the PLQY. Additionally, the smaller average size of the target QDs accounted for their blue-shifted emission. We fitted the time-resolved photoluminescence (TRPL) curves by using a biexponential equation (Fig. 2f and Table S1). The average carrier lifetimes (*τ*_avg_) of the pristine and target QD solutions were calculated to be 37.0 ns and 49.2 ns, respectively. The prolonged *τ*_avg_ indicated that the ion pairs effectively passivated the target QDs, achieving a lower defect density. We also monitored the stability of the QD solutions (Fig. 2g). The supernatant of the pristine QD solution exhibited an obvious decrease in photoluminescence (PL) intensity within 30 min, accompanied by substantial QD precipitation. After 60 min, the PL intensity had decreased to 39% of its initial value. In contrast, the target QD solution exhibited nearly constant PL intensity over 60 min. These results indicated that ion‑pair pinning would effectively suppress the aggregation and precipitation of QDs in the colloidal solution, thereby enhancing the stability of the QD solution.

### Ion-pair pinning effect on air-processed QD films

**Fig. 3:**
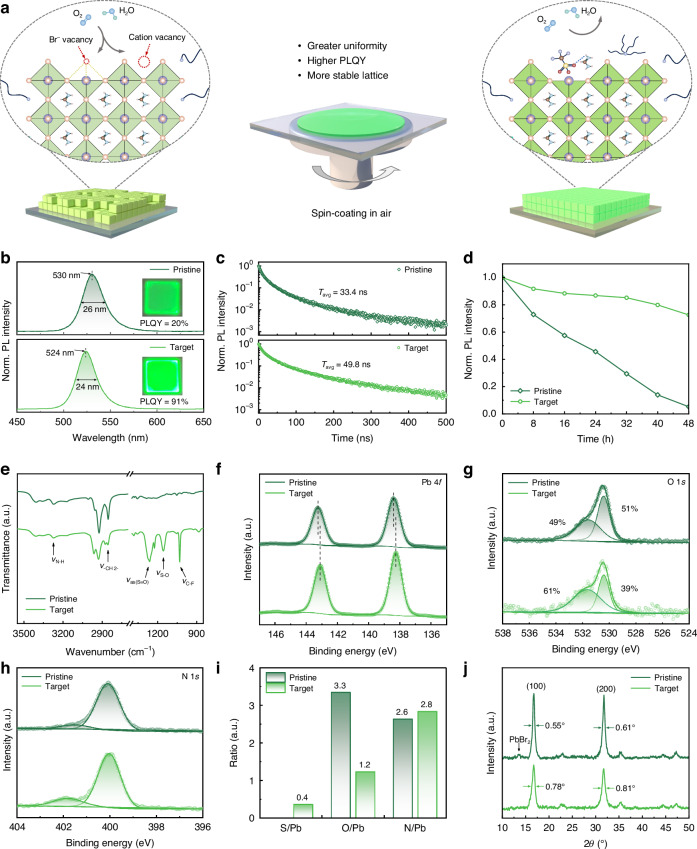
Ion-pair pinning effect on the air-processed QD films. **a** Schematic illustration of the ion-pair pinning effect on air-processed QD films. **b** PL spectra with the inset of photographs of the QD films under 365 nm UV excitation light. **c** TRPL curves. **d** PL stability of the QD films under 50 – 60% relative humidity. **e** FTIR spectra. HRXPS spectra of **f** Pb 4 *f*, **g** O 1 *s*, and **h** N 1 *s*. **i** Quantitative XPS analysis. **j** XRD patterns

These surface pinning effects are expected to enhance the QDs’ resistance to moisture and oxygen, enabling the preparation of high-quality QD films in ambient air (Fig. 3a). We used the QD solutions to fabricate thin films in ambient air and investigated the properties of the resulting QD films. Compared to the corresponding QD solutions, the resulting films both exhibited an emission redshift (5 nm for the pristine and 1 nm for the target), potentially attributed to the ripening of QDs during the spin-coating process (Fig. 3b). In addition to the smaller redshift, the target QD films exhibited narrower PL spectra and higher PLQY across all humidity fabrication conditions, indicating enhanced robustness of the target QDs (Fig. S4). We also measured TRPL curves to investigate the exciton dynamics in QD films. Compared with the pristine one, the target QD film showed a longer *τ*_avg_, a higher radiative decay rate (*K*_r_), and a lower nonradiative decay rate (*K*_nr_), indicating suppressed exciton quenching induced by defects (Fig. 3c and Table S2). In addition, after storage in ambient air with high humidity (50 ~ 60% relative humidity) for 48 h, the PL intensity of the pristine QD film dropped obviously to only 5% of its initial value (Fig. 3d). This could be attributed to the poor crystalline quality and higher defect density of the pristine QDs (Fig. 3b, c), which provided more sites for moisture and oxygen invasion. In contrast, the target QD film retained 73% of its initial PL intensity under the same conditions. The enhanced optical properties and stability were ascribed to the ion-pair pinning effect of TBAOTf: on the one hand, the ion pairs could improve the QD quality through crystallisation regulation; on the other hand, TBA^+^ and OTf^−^ could act as ligands which would suppress the loss of FA^+^ through multiple interactions and stabilise the exposed Pb^2+^ ions, enhancing the moisture-oxygen resistance of the QD films.

We performed FTIR and high-resolution X-ray photoelectron spectroscopy (HRXPS) to analyse the surface chemical states of the air-processed QD films. The FTIR spectra of the target QD film showed stretching vibrational peaks of S = O, S-O, and C-F bonds, confirming the pinning effect of OTf^−^ on the QD surface (Fig. 3e). The larger steric hindrance and lower structural symmetry of TBA^+^ caused a splitting of the -CH_2_- stretching vibrational peak at 2860 cm^−1^. These spectral alterations provided evidence for the pinning effect of TBA^+^ on the QD surface as an X-type ligand. The HRXPS results are shown in Fig. 3f–h and Fig. S5. The appearance of the S 2*p* peak in the target QD film proved that TBAOTf could attach to the QD surface. Compared to the pristine QD film, the Pb 4 *f* peaks and Br 3 *d* peaks shifted to lower binding energy in the target QD film. The increased electron cloud density around Pb and Br atoms originated from the passivated Pb^2+^ defects and more stable interactions between FA^+^ and QD lattice^25,26^. The narrower N 1 *s* peak at about 400.0 eV (from FA^+^) in the target QD film could be attributed to the improved uniformity of the chemical environment around N atoms, indicating enhanced QD size homogeneity and reduced surface FA^+^ defects. In the O 1 *s* core level spectrum, the signal at 530.5 eV originated from the oxidation of surface-exposed Pb^2+^ by ambient oxygen or residual moisture. For the target QD film, the component with low binding energy decreased. Combined with quantitative element analysis, the oxidation component induced by water and oxygen decreased by 3.3 times (Fig. 3i). Furthermore, the ultraviolet photoelectron spectroscopy (UPS) revealed a p-type transition in the target QD film, indicating the shallow defect states caused by Br vacancies have been suppressed (Fig. S6)^27^. From the above results, we concluded that TBAOTf could attach to the surface of QDs, strongly interact with FA^+^ and uncoordinated Pb^2+^. This reduced surface defects, increased the uniformity of QDs, and enhanced the moisture-oxygen resistance of QDs.

We used X-ray diffraction (XRD) to characterise the crystal structure of the QD films (Fig. 3j). For the pristine QD film, the diffraction peaks at 14.7° and 29.7° corresponded to the (100) and (200) crystal planes of the cubic phase, respectively^28^. For the target QD film, these diffraction peaks did not shift, suggesting that TBAOTf did not alter the lattice parameters. This finding was consistent with high-definition transmission electron microscopy (HRTEM) (Fig. S3). However, the broadening of the main diffraction peaks suggested that TBAOTf suppressed the growth of QDs during spin coating in ambient air^29^. Besides, we observed a weak PbBr_2_ characteristic peak at 13.7° in the pristine QD film, attributed to the moisture-oxygen-induced degradation of QDs, whereas no such peak was detected in the target film^30^. Surface chemical states and structural information of QDs revealed the surface pinning effects of TBAOTf, which could enhance QD stability during film formation and thereby suppress defects and undesired growth.

To understand the film-forming properties of the QD solutions during air processing, we conducted microstructural characterisation of the air-processed QD films. As shown in the confocal laser scanning fluorescence microscopy (CLSFM) images, the pristine QD film exhibited disordered PL signal distribution, with obvious bright spots and dark regions (Fig. S7a), indicating serious QD aggregation and defects derived from the accelerated ripening of QDs^18^. By comparison, the target QD film exhibited continuous, uniform PL signals, demonstrating that the synergistic ion-pairing pinning strategy could effectively inhibit QD ripening-induced aggregation (Fig. S7b). Scanning electron microscope (SEM) and atomic force microscopy (AFM) characterisations corroborated the aforementioned conclusion. The pristine QD film showed high inhomogeneity, with a root-mean-square (RMS) roughness of 8.3 ± 1.2 nm and discrete dark regions (Figs. S8a, S9a), which would lead to severe leakage currents in the QD-based optoelectronic devices^31^. The high-magnification SEM image further revealed the loose structure of these hole-like defects, resulting in rough surfaces. In contrast, both the SEM and AFM images of the target QD film exhibited homogeneous morphology with a RMS roughness of 4.9 ± 1.1 nm, showing no obvious aggregation or dark areas (Figs. S8b, S9b). The above results demonstrated that TBAOTf could effectively suppress moisture-oxygen-induced defects and QD aggregation during air processing, providing a foundation for the superior carrier-transport properties and optoelectronic performance of the QD films.

**Fig. 4:**
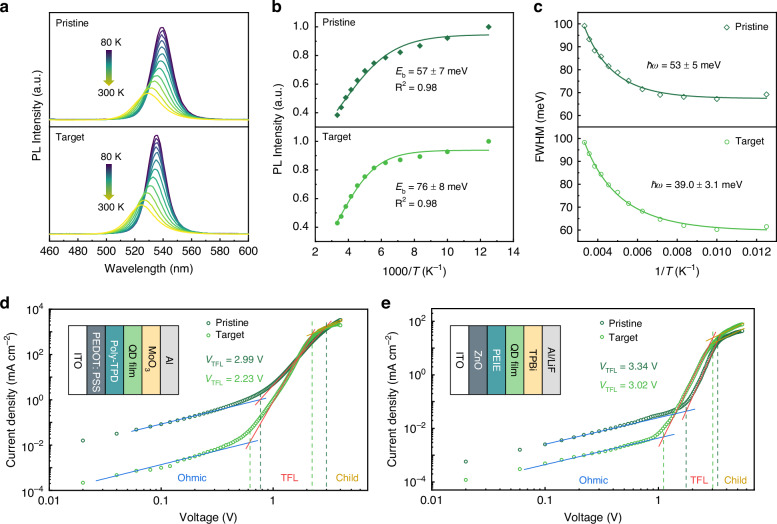
The exciton behaviour and trap state of the air-processed QD films. **a** Temperature-dependent PL spectra. **b** Integrated PL intensity and **c** FWHM as a function of the reciprocal temperature. Current density-voltage curves and schematic structures of the **d** hole-only and **e** electron-only devices

We then analysed the exciton behaviour of the air-processed QD films. Temperature-dependent PL spectra demonstrated that both the pristine and target QD films exhibited a blue shift in PL peak with increasing temperature (Fig. 4a). Combined with the characteristic of multi-effect synergy in the temperature dependence of QD bandgap, this temperature-dependent PL blue shift phenomenon is mainly attributed to the dominant role of intraband electron-phonon coupling and mechanical strain effects^32^. The exciton binding energy (*E*_b_) of the pristine and target QD films determined from the Arrhenius fitting of temperature-dependent PL intensity showed the value of 57 ± 7 meV and 76 ± 8 meV, respectively (Fig. 4b). The increased *E*_b_ of the target QD film, reflecting the strengthened lattice by ion-pair pinning, contributed to suppressed exciton dissociation and enhanced radiative recombination^12^. Further, we fitted the temperature dependence of the FWHM by using the following equation^33^:

1 $$\mathrm{FWHM}={\varGamma }_{0}+\frac{{\varGamma }_{{\boldsymbol{L}}0}}{\exp (\frac{\hslash \omega }{{k}_{{\boldsymbol{B}}}T}-1)}$$

where *Г*_0_ is the inhomogeneous broadening term, *Γ*_LO_ is the Fröhlich coupling coefficient, *k*_B_ is the Boltzmann constant, *T* is the absolute temperature, and ℏ*ω* represents the electron-optical phonon coupling strength induced by defects and lattice disorder (ℏ is the Planck constant, and *ω* is the frequency of the longitudinal optical phonon vibration). Notably, the target QD film (ℏ*ω* = 39.0 ± 3.1 meV) exhibited a lower electron-phonon coupling strength than the target film (ℏ*ω* = 53 ± 5 meV) did, suggesting that the ion-pair pinning effect could stabilise the lattice and reduce exciton scattering centres, thereby suppressing defect-induced non-radiative relaxation (Fig. 4c).

Subsequently, we employed the space-charge-limited current (SCLC) model in hole-only and electron-only devices to evaluate the trap state density. The hole-only device structure was indium tin oxide (ITO)/ poly(3,4-ethylenedioxythiophene): poly(styrenesulfonate) (PEDOT: PSS)/ poly(N,N’-bis(4-butylphenyl)-N,N’-bis(phenyl)-benzidine) (poly-TPD)/ QD film/ molybdenum oxide (MoO_x_)/ aluminium (Al), and the electron-only device structure was ITO/ zinc oxide (ZnO)/ polyethylenimine ethoxylated (PEIE)/ QD film/ 1,3,5-tris(1-phenyl-1H-benzimidazol-2-yl)benzene (TPBi)/ LiF/ Al. The current density-voltage curves of the pristine and target devices are depicted in Fig. 4d, e. The trap state density of the QD films could be calculated by using the equation^4^:

2 $${N}_{{\rm{t}}}=\frac{2{\varepsilon }_{0}{\varepsilon }_{r}{V}_{{\rm{TFL}}}}{e{L}^{2}}$$

where *N*_t_ is the trap state density, *V*_TFL_ is the trap-filled limit voltage, *e* is the elementary charge, *ε*_0_ is the vacuum permittivity, *ε*_r_ is the relative permittivity (*ε*_r_ = 43.6 for FAPbBr_3_^34^), and *L* is the thickness of the QD films. The results from both hole-only and electron-only devices indicated that the target QD films exhibited lower *V*_TFL_ than the pristine ones did. The *N*_t_ of the target QD films were determined to be 1.19 × 10^19^ cm^−3^ and 1.62 × 10^19^ cm^−3^ in the hole-only and electron-only devices, respectively, both lower than those of the pristine QD films. The decreases in *N*_t_ indicated that TBAOTf would effectively suppress defect generation, which was consistent with the previously observed improvements in the optical performance and stability of the QDs. These results validated that the ion-pair pinning effects of TBAOTf, which would enable the construction of high-efficiency QLEDs in ambient air.

### Device performance

**Fig. 5:**
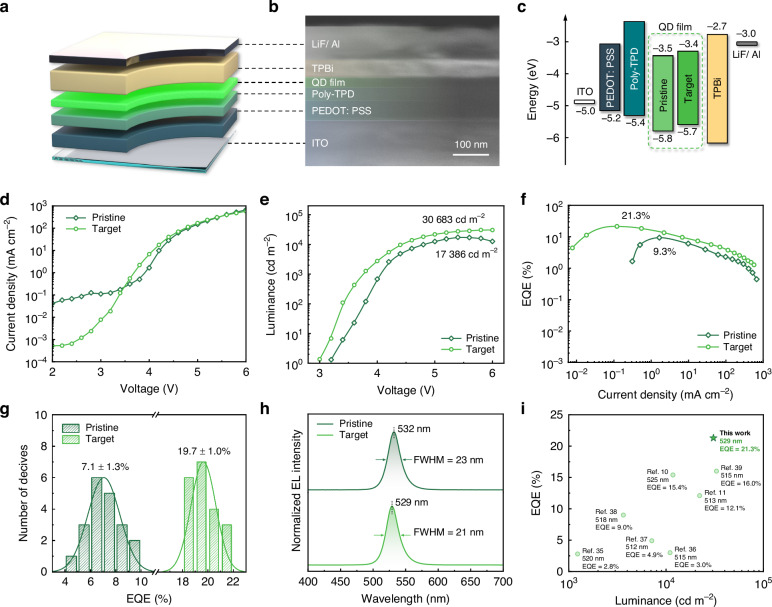
Air-processed QLED performance. **a** Schematic structure, **b** cross-sectional SEM image, and **c** energy diagram of the device. **d** Current density-voltage curves. **e** Luminance-voltage curves. **f** EQE-current density curves. **g** Statistical EQEs of 20 devices. **h** EL spectra. **i** Summary of the reported air-processed green perovskite LEDs

To evaluate the EL properties of these air-processed QD films, we finally constructed perovskite QLEDs with a multi-layered structure of ITO/ PEDOT: PSS (45 nm)/ poly-TPD (45 nm)/ QD film (30 nm)/ TPBi (40 nm)/ LiF (1 nm)/ Al (100 nm) (Fig. 5a, b). The energy levels of the QD films were measured by using UPS (Figure S6), enabling the aligned energy diagram (Fig. 5c). We measured the current density-voltage curves of the pristine and target devices (Fig. 5d) and found that the pristine device exhibited severe current leakage in the initial driving voltage range (from 2 V to 3 V), probably attributed to the hole-like defects. For the target device, the current leakage was effectively suppressed, resulting in a lower turn-on voltage of 3.0 V (compared to 3.2 V for the pristine QLED) (Fig. 5e).

Benefiting from the comprehensive optimisation of the air-processed QD films enabled by the ion-pair pinning strategy, the target devices achieved superior performance compared to the pristine devices across all humidity fabrication conditions (Fig. S10). Under ambient conditions at 20% relative humidity, the target device realised a maximum luminance of 30,683 cd m^−2^, a peak EQE of 21.3%, an EQE of 15.0% at 1000 cd m^−2^, and showed good reproducibility with an average EQE of 19.7 ± 1.0% of 20 devices (Fig. 5e–g). The EL spectra of the devices at the peak luminance are shown in Fig. 5h. The target device exhibited a slight blue shift in the EL peak wavelength compared to the pristine one, with a smaller FWHM. Consequently, the corresponding CIE chromaticity coordinates of the target device shifted from (0.21, 0.75) to (0.19, 0.76), and the latter was closer to the Rec. 2020 standard of (0.17, 0.80) (Fig. S11). We also evaluated the operating stability of the QLEDs and found that, compared to the pristine one, the target device showed nearly 6.5 times longer *T*_50_ at an initial luminance of 100 cd m^−2^ (Fig. S12). Overall, by using this ion-pair pinning strategy, we have achieved the best performance reported to date for air-processed green perovskite LEDs, even comparable to that of N_2_-processed perovskite QLEDs (Fig. 5i and Table S3)^10,11,35–39^.

**Fig. 6:**
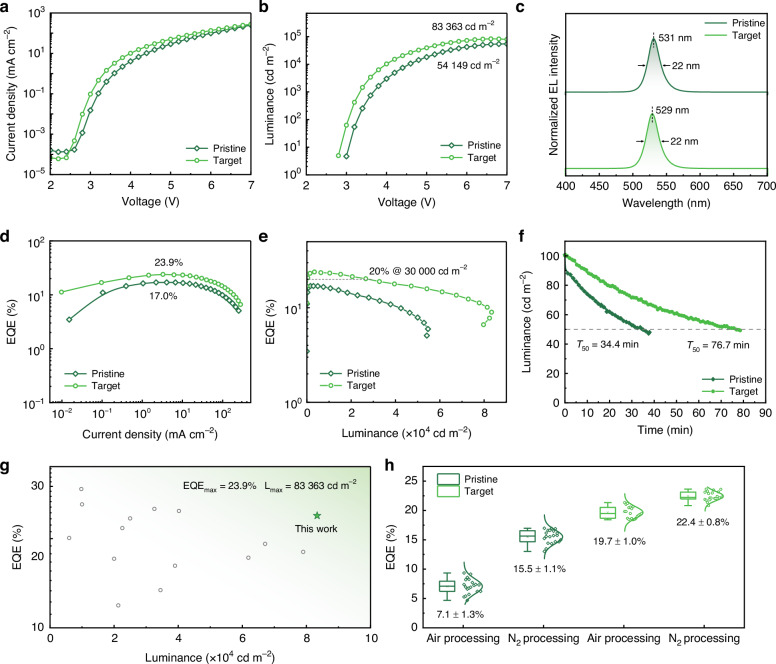
N_2_-processed QLED performance. **a** Current density-voltage curves. **b** Luminance-voltage curves. **c** EL spectra. **d** EQE-current density curves. **e** EQE-luminance curves. **f** Operating stability. **g** Summary of the reported FAPbBr_3_-based QLEDs. **h** Statistical EQEs of the pristine and target devices under air and N_2_ fabrication conditions

For comparison, we further constructed N_2_-processed QLEDs. Under N_2_ protection, neither the pristine nor the target QLED exhibited current leakage at low voltages (Fig. 6a). The target device achieved an ultra-high peak luminance of 83,363 cd m^−2^ and a maximum EQE of 23.9%, compared to 54,149 cd m^−2^ and 17.0% for the pristine device, respectively (Fig. 6b–d). More importantly, the target device maintained a high EQE of 20% even at a luminance of 30,000 cd m^−2^ (Fig. 6e). Furthermore, the *T*_50_ of the target device at an initial luminance of 100 cd m^−2^ increased from 34.4 min in the original group to 76.7 min (Fig. 6f). Benefiting from the suppressed efficiency roll-off and enhanced stability enabled by the ion-pair pinning strategy, we have realised the record peak luminance of FAPbBr_3_-based QLEDs reported to date (Fig. 6g and Table S4). Subsequently, we statistically analysed the EQE distribution of air-processed and N_2_-processed QLEDs. As shown in Fig. 6h, the average EQE of the pristine N_2_-processed QLEDs increased to 15.5%, more than doubling that of air-processed QLEDs (7.1%). Meanwhile, the target devices exhibited an increase in average EQE from 19.7% for air-processed QLEDs to 22.5% for N_2_-processed ones. These results consistently demonstrated that the N_2_ fabrication conditions greatly enhanced the overall performance of the QLEDs. Moreover, they confirmed the universal effectiveness of the proposed ion-pair pinning strategy for optimising QDs under N_2_ conditions, while also highlighting its even more pronounced benefits under ambient fabrication conditions. This finding underlined the practical importance of the proposed strategy for ambient processing of QDs, providing strong support for expanding QD application prospects in ambient air.

## Discussion

In summary, we developed an ion-pair pinning strategy by adding TBAOTf into the perovskite precursor solutions to enhance the optoelectronic performance of air-processed FAPbBr_3_ QD films. Theoretical calculations and experimental results suggested that TBAOTf could influence the crystallisation of FAPbBr_3_ QDs through multiple interactions and act as X-type ligands to stabilise the QD surface states, thereby enhancing the PL properties and stability of the QD solution. Additionally, the pinned TBAOTf could suppress defect formation during the air processing of QD films, thereby improving the film quality. Therefore, we achieved high-performance pure-green perovskite QLEDs processed in both air and N_2_ conditions. The air-processed device showed a maximum EQE of 21.3% and a peak luminance of 30,683 cd m^−2^, representing the best performance of air-processed green perovskite LEDs. Meanwhile, the N_2_-processed one reached a high EQE of 23.9% and an ultra-high peak luminance of 83,363 cd m^−2^. We expect that this work would inspire more researchers to focus on and address the stability issue caused by the moisture-oxygen sensitivity of perovskite materials, thereby developing perovskite inks with low-cost fabrication, excellent optoelectronic properties, and scalable applications.

## Materials and Methods

### Materials

Formamidine bromide (FABr, 99.5%), N,N-dimethylformamide (DMF, 99.8%), lead bromide (PbBr_2_, 99.99%), poly(N,N’-bis(4-butylphenyl)-N,N’-bis(phenyl)-benzidine) (poly-TPD), 1,3,5-Tris(1-phenyl-1H-benzimidazol-2-yl)benzene (TPBi), and poly(3,4-ethylenedioxythiophene)-poly(styrenesulfonate) (PEDOT: PSS, AI 4083) were purchased from Xi’an Yuri Solar Co., Ltd. Chloroform was purchased from Sinopharm Chemical Reagent Co., Ltd. Ammonium bromide (NH_4_Br, 99%), tetraethylammonium bromide (TEABr, 99%), tetrabutylammonium bromide (TBABr, 99%), ammonium triflate (NH_4_OTf, 99%), tetrabutylammonium triflate (TBAOTf, 98%), *n*-octane (99%), *n*-octylamine (OTAm, 99%), oleic acid (OA, 85%), and acetonitrile (99.9%) were purchased from Aladdin Reagent Co., Ltd. Ammonium methanesulfonate (NH_4_MeS, 98%) was purchased from J&K Scientific. All chemicals were utilised as received without further purification.

### Synthesis and purification of FAPbBr_3_ QDs

FAPbBr_3_ QDs were synthesised under ambient conditions (25 ± 2 °C, 25% relative humidity). First, precursor solutions were prepared by mixing FABr (0.2 mmol), PbBr_2_ (0.1 mmol), OA (250 μL), and OTAm (25 μL) in 0.5 mL DMF. The precursor solution was then rapidly injected into 8 mL chloroform with vigorous stirring. After 35 s, acetonitrile was added to the crude QD solution at a volume ratio of 3:1 and centrifuged at 10000 r.p.m. for 5 min. The QD precipitate was collected then dispersed into *n*-octane and centrifuged again at 6000 r.p.m. for 3 min. The resulting supernatant was collected to obtain the FAPbBr_3_ QDs solution. The synthesis and purification of FAPbBr_3_ QDs modified with ion pairs were identical to those described above, differing only in the addition of different ion pairs to the precursor mixture.

### Device fabrication

Unless otherwise specified, the following processes were conducted at 25 ± 2 °C and different relative humidity levels (about 20%, 40%, and 60%) in ambient air. The patterned ITO was treated with de-ionised water, acetone, and ethyl alcohol by ultrasonic treatment for 10 min and then treated with oxygen plasma for 4 min before use. PEDOT:PSS was filtered by a 0.22 μm filter and then coated on an ITO-coated glass substrate at 4000 r.p.m. for 45 s and heated at 150 °C for 20 min. A solution of poly-TPD in chlorobenzene (5 mg mL^−1^) was spin-coated at 4000 r.p.m. for 45 s and baked at 120 °C for 20 min. Then, the solution of FAPbBr_3_ QDs was spin-coated at 1000 r.p.m. for 45 s. Finally, TPBi (40 nm) and LiF/ Al electrodes (1 nm/ 100 nm) were deposited at different rates successively under high vacuum using a thermal evaporation system.

### Characterisation

The ^1^H NMR spectra were acquired with Bruker AV-HD-400X. FTIR of precursor and QD films obtained using Horiba VERTEX 70 with ATR mode. XPS and UPS of the QD films were characterised by using an ESCALAB Xi+ spectrometer. XRD patterns were recorded by using a Bruker D8 Advance X-ray diffractometer with Cu *K*_α_ radiation (*λ* = 1.5406 Å), and samples for XRD measurements were prepared by spin-coating purified QD solutions on glass substrates. HRTEM images of the QDs were obtained by using a JEM2010 transmission electron microscope operating at 200 kV. HRTEM sample preparation involved drop-casting diluted synthetic QD dispersion onto carbon-coated copper grids, followed by evaporation of *n*-octane at room temperature. The QD solutions were spin-coated onto Si substrates, and a Veeco D3100 AFM tool was utilised to assess film roughness and surface current mapping. CLSFM images of QD films obtained by using Olymplus FV1200. SEM images of the QD films and device cross-sections were acquired by using a HITACHI SU-8010 cold-field emission scanning electron microscope. The absorption spectra of the QD solutions were measured by using a Shimadzu UV-3600 UV/VIS/NIR spectrophotometer. The PL spectra of the QD solutions were acquired with a Varian Cary Eclipse spectrometer, and PL decay was measured by using a time-correlated single-photon counting (TCSPC) spectrofluorometer (FLS920, Edinburgh Instruments, UK). Temperature-dependent PL spectra and PL decay were recorded on a JY-U1000 spectrometer equipped with an LN2-cooled CCD camera.

### Device evaluation

The effective light-emitting area of the device was 3 mm^2^ as defined by the overlapping area of the ITO and Al electrodes. We employed the method of Forrest et al. to evaluate the QLEDs at room temperature in a N_2_-filled glovebox^40^. The current density-voltage characteristics were measured by using a source metre (Keithley 2400, Tektronix), and the voltage was swept from 2 to 6 V in 0.2 V steps with an integration time of 200 ms. The EL characteristics were measured by using a fibre integration sphere and an Ocean Optics USB4000 spectrometer. The stability test was conducted under the same environmental conditions and with the identical equipment to ensure consistency and comparability. The devices were driven in the constant current mode of the source metre, with the initial luminance calibrated to 100 cd m^–2^, while the EL intensity was monitored in real time. The system was calibrated by using a radiometric-calibrated light source (HL-3P-INT-CAL, Ocean Optics) with reference spectral radiant flux. The QLEDs were mounted on the open aperture of the integrating sphere to allow the light emitted from the glass surface to be collected, while the emission from the substrate edges was not collected. A Lambertian emission profile was assumed when calculating the luminance.

### Computational details

First-principles calculations based on DFT were performed using the Vienna Ab initio Simulation Package (VASP)^41^. The Perdew–Burke–Ernzerhof (PBE) functional^42^ was employed for the exchange-correlation interactions, and the DFT-D3 method was applied to account for van der Waals (vdW) corrections^43^. A plane-wave energy cutoff of 400 eV was used. The convergence criteria for energy and force were set to 10^−5^ eV and 0.03 eV Å^−1^, respectively. AIMD simulations were carried out by using the CP2K package^44^. We constructed the model for the perovskite precursor solution with a mixture of ions and molecules: 12 Pb^2+^, 36 Br^−^, 12 FA^+^, 2 OTf^−^, 4 DMF, and 2 TBA^+^. In the initial configuration, all species were randomly distributed by using the Packmol software^45^. The AIMD simulations were first equilibrated for 9 ps in the constant-pressure and constant-temperature (NPT) ensemble, followed by 10 ps in the constant-volume and constant-temperature (NVT) ensemble. Temperature was maintained at 300 K using the Nosé-Hoover thermostat^46^, and pressure was set to 1 bar. The time step was 1.0 fs. The PBE-D3 functional was used with double-zeta basis sets (DZVP-MOLOPT)^47^ and Goedecker–Teter–Hutter (GTH) pseudopotentials^48^, with a plane-wave cutoff of 400 Ry.

## Supplementary information

Supporting Information

## Data Availability

The data that support the findings of this study are available from the corresponding author upon reasonable request.
