# Supplementary material for: Ion-pair pinning on perovskite quantum dots for high-efficiency air-processed light-emitting diodes with Rec. 2020 compliance
Source: Light Sci Appl. 2026 Mar 6;15:151. doi: 10.1038/s41377-026-02247-z (PMC12966369; doi:10.1038/s41377-026-02247-z)
Supplement: Supplementary file 1 — Supporting Information [file 41377_2026_2247_MOESM1_ESM.pdf]

Supporting Information for

Ion-pair pinning on perovskite quantum dots for high-efficiency air-processed light-emitting diodes with Rec. 2020 compliance

Yuhang Cui[1], Danlei Zhu[1], Jiawei Chen[1,2,*], Shuyue Dong[1], Yuanzhuang Cheng[1], Xiangyu Liu[1], Xinghua Yan[1], Zicong Jin[1], Lian Duan[1,3], Jian Xu[4,*], Dongxin Ma[1,3,*]

[1]*Key Lab of Organic Optoelectronics and Molecular Engineering of Ministry of Education, Department of Chemistry, Tsinghua University, Beijing, 100084, China.*

[2]*MIIT Key Laboratory of Advanced Display Materials and Devices, School of Materials Science and Engineering, Nanjing University of Science and Technology, Nanjing, 210094, China.*

[3]*State Key Laboratory of Flexible Electronics Technology, Tsinghua University, Beijing, 100084, China.*

[4]*School of Interdisciplinary Science, Beijing Institute of Technology, Beijing 100081, China*

Email: chenjiawei@njust.edu.cn; jianxu.xu@bit.edu.cn; dongxinma@tsinghua.edu.cn

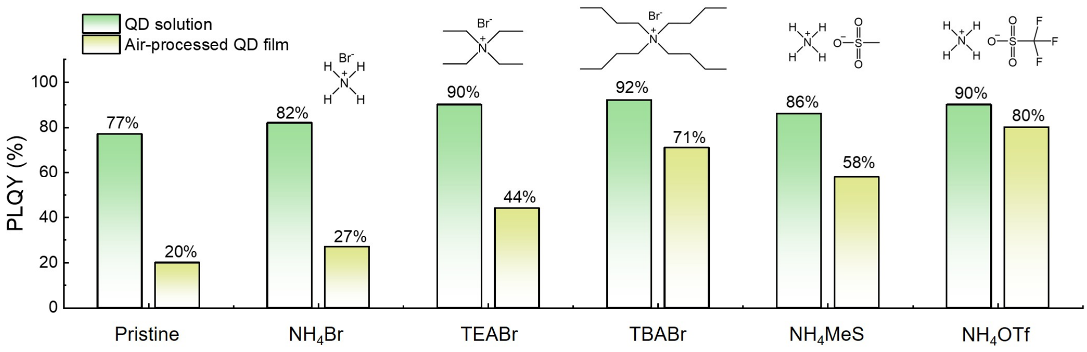

Fig. S1 Comparison of the photoluminescence quantum yield (PLQY) of quantum dot (QD) solutions and air-processed films, which were treated with different ion pairs (4 mol% Pb^{2+}).

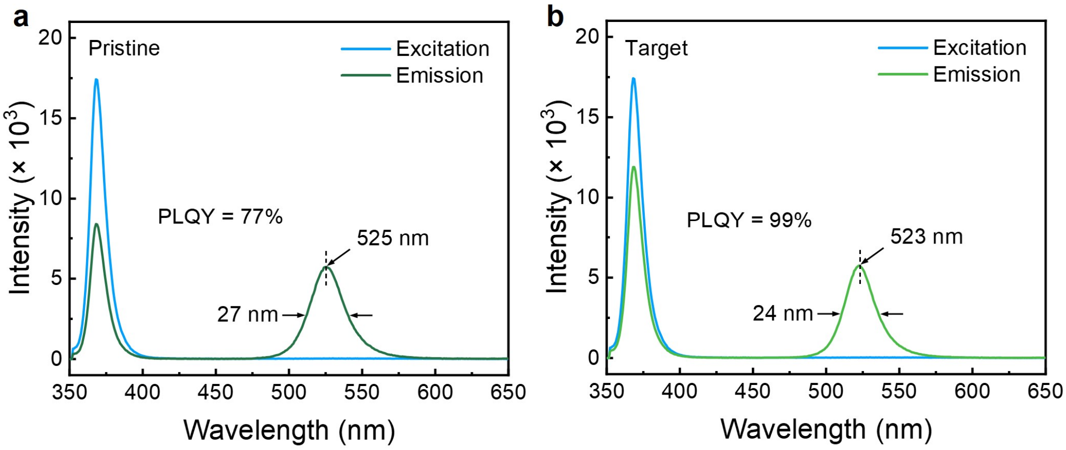

Fig. S2 PLQY spectra of the (a) pristine and (b) target QD solutions.

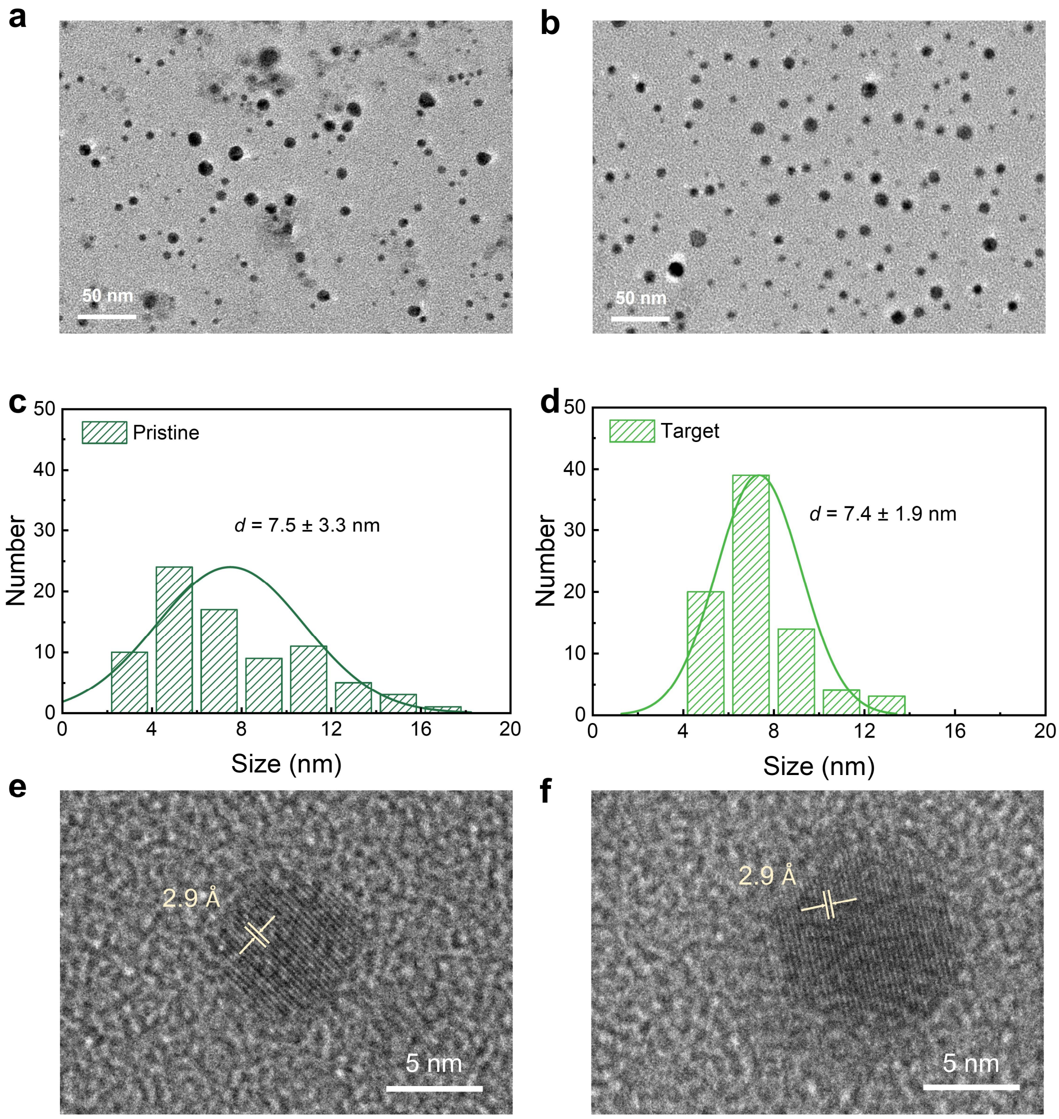

Fig. S3 Transmission electron microscopy (TEM) images of the (a) pristine and (b) target QDs. The size distribution histograms of the (c) pristine and (d) target QDs. High-resolution transmission electron microscopy (HRTEM) images of the (e) pristine and (f) target QDs.

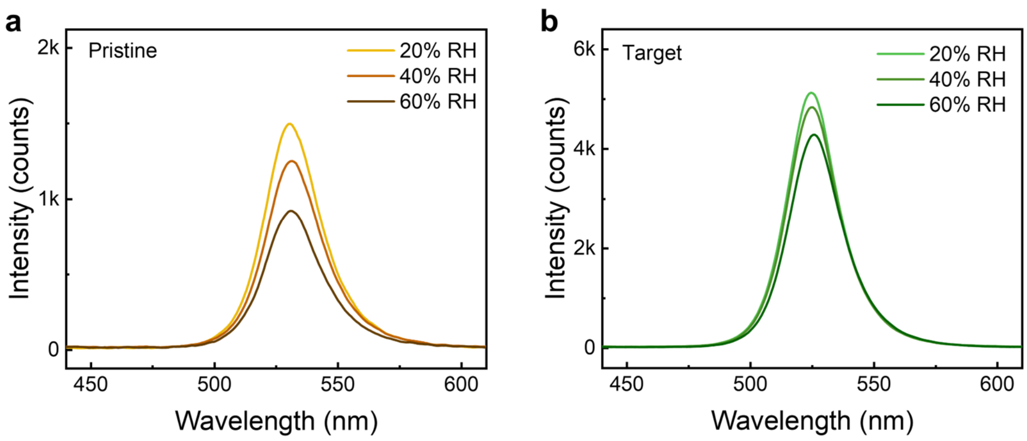

Fig. S4 Photoluminescence (PL) spectra of the (a) pristine and (b) target QD films under different relative humidity (RH) fabrication conditions.

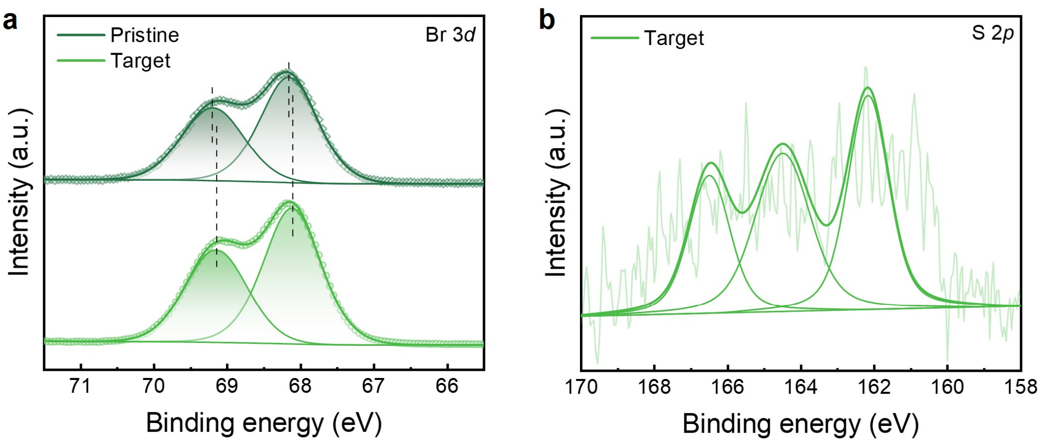

Fig. S5 High-resolution X-ray photoelectron spectroscopy (HRXPS) spectra of (a) Br 3*d* and (b) S 2*p* of air-processed QD films.

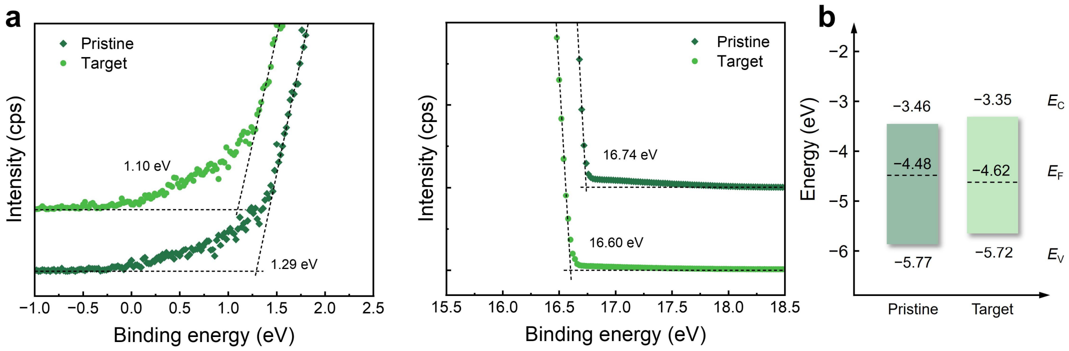

Fig. S6 (a) Ultraviolet photoelectron spectroscopy (UPS) of the pristine and target QD films. (b) Energy band diagram.

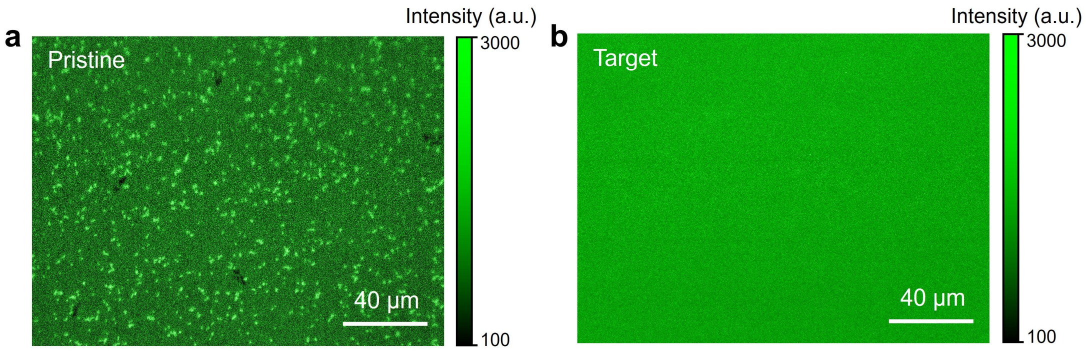

Fig. S7 Confocal laser scanning fluorescence microscopy (CLSFM) images of the (a) pristine and (b) target QD films.

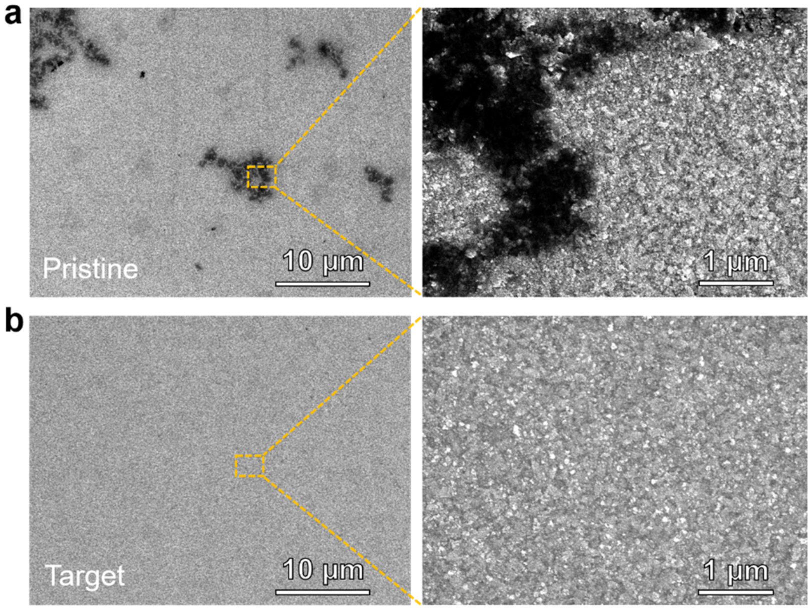

Fig. S8 Scanning electron microscopy (SEM) images of the (a) pristine and (b) target QD films at different magnifications.

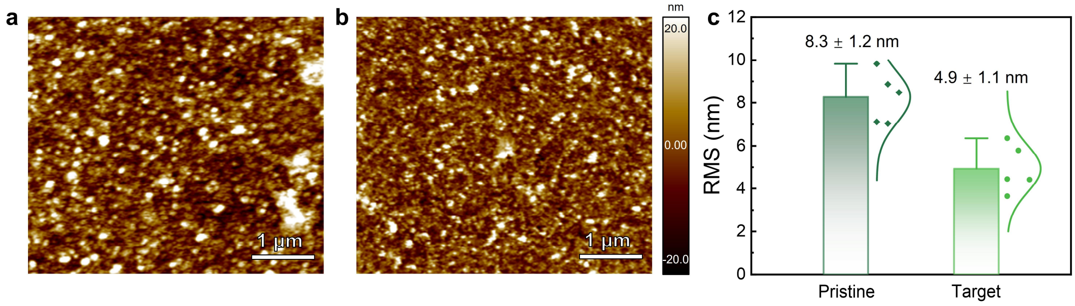

Fig. S9 Typical atomic force microscopy (AFM) images of the (a) pristine and (b) target QD films. (c) The statistical root-mean-square (RMS) roughness of 5 samples.

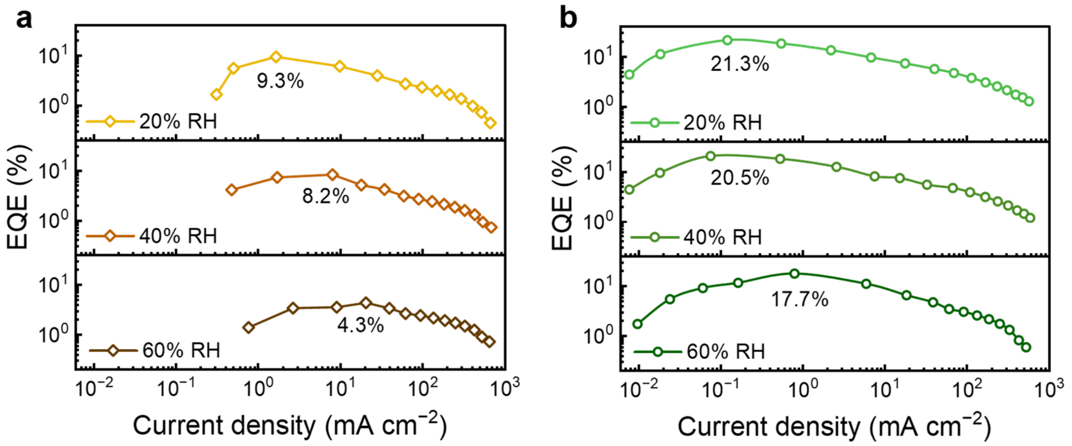

Fig. S10 EQE curves of the (a) pristine and (b) target QLEDs under different RH fabrication conditions.

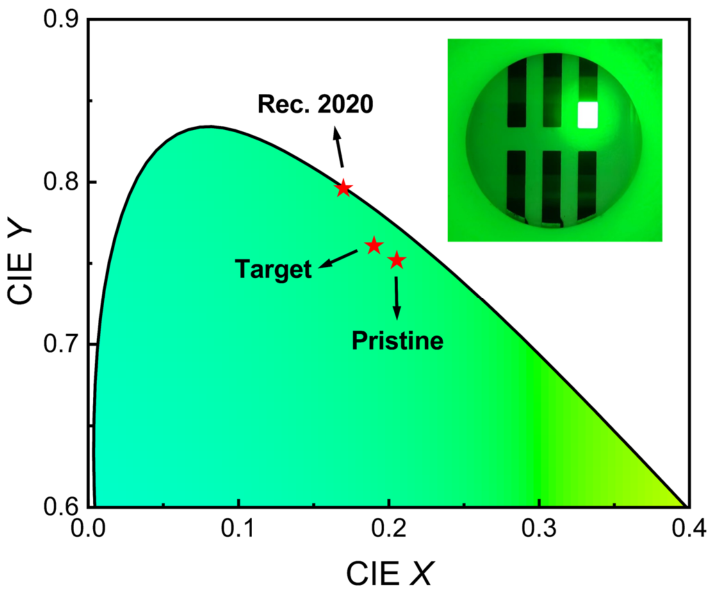

Fig. S11 Commission Internationale de l'Eclairage (CIE) chromaticity diagram with an inset showing a photograph of the working target QLED at 5.8 V.

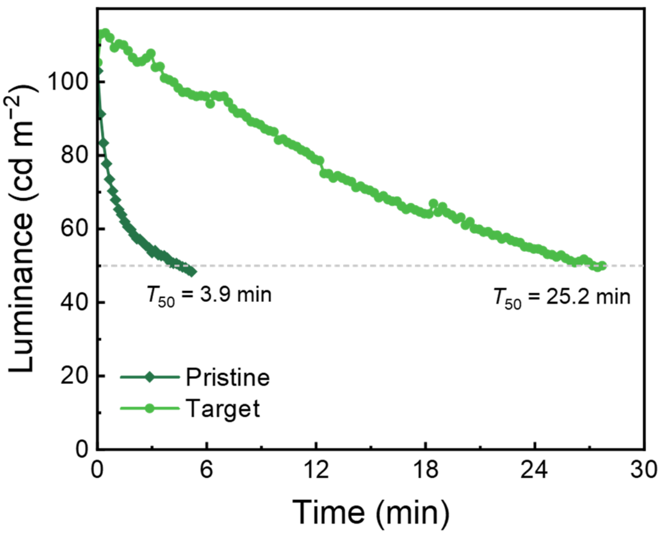

Fig. S12 Operating stability of the air-processed QLEDs with an initial luminance of 100 cd m^{-2}.

Table S1. Summary of parameters for the time-resolved PL decay curves of the QD solutions.

Sample	A_1	τ_1 (ns)	A_2	τ_2 (ns)	τ_{avg} (ns)
Pristine	0.65	9.24	0.35	47.03	37.04
Target	0.69	10.23	0.31	63.33	49.22

The τ_1 originated from surface-related recombination and τ_2 originated from intrinsic recombination[2]. The average decay time (τ_{avg}) was calculated by using a weighted average formula derived from the bi-exponential fitting parameters, as described in **Equation 3**:

$$\tau_{avg} = \frac{A_1\tau_1^2 + A_2\tau_2^2}{A_1\tau_1 + A_2\tau_2} \quad (3)$$

Table S2. Summary of parameters for the time-resolved PL decay curves of the QD films.

**Sample**	A_1	τ_1 (ns)	A_2	τ_2 (ns)	τ_{avg} (ns)	**PLQY (%)**	K_r (10^6 s^{-1})	K_{nr} (10^6 s^{-1})
Pristine	0.69	7.54	0.31	43.29	33.41	20.37	6.10	23.83
Target	0.65	9.59	0.35	61.53	49.77	90.86	18.27	1.83

Radiative decay rate (K_r) represents the rate at which excitons return to the ground state by emitting photons. A higher K_r indicates stronger radiative recombination in the system. Non-radiative decay rate (K_{nr}) denotes the rate at which excitons transition to the ground state by releasing energy through non-photonic pathways. A larger K_{nr} suggests dominant non-radiative recombination processes. K_r and K_{nr} were calculated from the PLQY and τ_{avg} values by using **Equations 4** and **5**:

$$\mathrm{PLQY} = \frac{K_r}{K_r + K_{nr}} \tag{4}$$

$$K_r + K_{nr} = \frac{1}{\tau_{avg}} \tag{5}$$

Table S3. Summarized performance of the reported air-processed green perovskite LEDs.

Emitters	EQE (%)	Peak luminance (cd m^{-2})	EL peak (nm)	Ref.
Quasi-2D perovskites	16.0	32,934	515	3
Quasi-2D perovskites	15.4	11,765	525	4
Quasi-2D perovskites	12.1	22,121	513	5
Quasi-2D perovskites	9.0	3,640	518	6
Quasi-2D perovskites	4.9	7,143	512	7
Perovskite QDs	3.0	10,992	515	8
Perovskite QDs	2.8	1,233	520	9
**Perovskite QDs**	**21.3**	**30,683**	**529**	**This work**

Table S4. Summarized performance of the reported N_2-processed $FAPbBr_3$-based QLEDs.

EQE (%)	Peak luminance (cd m^{-2})	EQE @ 30 000 cd m^{-2} (%)	Ref.
20.1	5,980	-	10
29.4	9,783	-	11
26.1	~10,000	-	12
17.1	~20,000	-	13
11.9	21,304	-	14
21.7	22,575	-	15
23.4	25,000	-	16
25.2	32,575	~10	17
13.4	34,480	~5	18
16.2	39,000	~10	19
24.8	40,231	~17	20
17.2	61,900	~10	21
19.2	67,115	~16	22
18.0	79,000	~13	23
**23.9**	**83,363**	**~20**	**This work**

Reference

1. Song, Y. et al. Efficient deep-blue LEDs based on colloidal $CsPbBr_3$ nanoplatelets meeting the Rec.2020 standard. *Light Sci. Appl.* **14**, 336 (2025).
2. Wu, Y. et al. In situ passivation of $PbBr_6^{4-}$ octahedra toward blue luminescent $CsPbBr_3$ nanoplatelets with near 100% absolute quantum yield. *ACS Energy Lett.* **3**, 2030-2037 (2018).
3. Tong, Y. et al. Regulating crystallization and carrier recombination of quasi-2D perovskite for efficient air-processed light-emitting diodes. *Nano Lett.* **25**, 8834-8842 (2025).
4. Liu, Y. et al. Phase aggregation suppression of homogeneous perovskites processed in ambient condition toward efficient light-emitting diodes. *Adv. Funct. Mater.* **31**, 2103399 (2021).
5. Li, W. et al. Reducing nonradiative losses of air-processed perovskite films via interface modification for bright and efficient light emitting diodes. *Adv. Funct. Mater.* **34**, 2311133 (2024).
6. Li, Y. et al. Coffee-stain-free perovskite film for efficient printed light-emitting diode. *Adv. Opt. Mater.* **9**, 2100553 (2021).
7. Li, W. et al. Fabrication of highly luminescent quasi two-dimensional $CsPbBr_3$ perovskite films in high humidity air for light-emitting diodes. *ACS Appl. Mater. Interfaces* **15**, 36602-36610 (2023).
8. Zheng, C. et al. High-brightness perovskite quantum dot light-emitting devices using inkjet printing. *Org. Electron.* **93**, 106168 (2021).
9. Li, D. et al. Inkjet printing matrix perovskite quantum dot light-emitting devices. *Adv. Mater. Technol.* **5**, 2000099 (2020).
10. Wang, H. et al. A multi-functional molecular modifier enabling efficient large-

area perovskite light-emitting diodes. *Joule* **4**, 1977-1987 (2020).

11. Zhang, J. et al. Fine-tuning crystal structures of lead bromide perovskite nanocrystals through trace cadmium (II) doping for efficient color-saturated green LEDs. *Angew. Chem. Int. Ed.* **63**, e202403996 (2024).

12. Kim, D.-H. et al. Surface-binding molecular multipods strengthen the halide perovskite lattice and boost luminescence. *Nat. Commun.* **15**, 6245 (2024).

13. Chen, H. et al. High-efficiency formamidinium lead bromide perovskite nanocrystal-based light-emitting diodes fabricated via a surface defect self-passivation strategy. *Adv. Optical Mater.* **8**, 1901390 (2020).

14. He, S. et al. Optical engineering of $FAPbBr_3$ nanocrystals via conjugated ligands for light-outcoupling enhancement in perovskite light-emitting diodes. *Adv. Optical Mater.* **11**, 2300486 (2023).

15. Wang, Y. et al. Quantum-confined perovskite nanocrystals enabled by negative catalyst strategy for efficient light-emitting diodes. *Small* **20**, 2402825 (2024).

16. Kim, Y.-H. et al. Comprehensive defect suppression in perovskite nanocrystals for high-efficiency light-emitting diodes. *Nat. Photonics* **15**, 148-155 (2021).

17. Lee, D. et al. Multisite coordination ligand strategy for $FAPbBr_3$ nanocrystal light-emitting diodes. *ACS Energy Lett.* **10**, 1411-1420 (2025).

18. Chin, X. et al. Self-assembled hierarchical nanostructured perovskites enable highly efficient LEDs via an energy cascade. *Energy Environ. Sci.* **11**, 1770-1778 (2018).

19. Zeng, Q. et al. Unveiling the effect of synthetic atmospheric humidity on the performance of $FAPbBr_3$ nanocrystals and their PeLEDs. *ACS Photonics* **12**, 429-438 (2025).

20. Zhang, J. et al. In-situ surface repair of $FAPbBr_3$ quantum dots toward high-

performance pure-green perovskite light-emitting diodes. *Nano Lett*. **24**, 12196-12203 (2024).

21. Wang, Q. et al. Drag force shear manipulating ligand distribution at perovskite buried interface enables efficiently suppressed EQE roll-off of perovskite light-emitting diodes. *Nano Energy* **127**, 109797 (2024).

22. Zhang, H. et al. High-brightness perovskite light-emitting diodes based on $FAPbBr_3$ nanocrystals with rationally designed aromatic ligands. *ACS Energy Lett.* **6**, 2395-2403 (2021).

23. Wang, Q. et al. Molecularly designing a passivation ETL to suppress EQE roll-off of PeLEDs. *ACS Energy Lett.* **8**, 3710-3719 (2023).
